# Genetic Analysis of Adult Plant Resistance to Stripe Rust in Common Wheat Cultivar “Pascal”

**DOI:** 10.3389/fpls.2022.918437

**Published:** 2022-07-06

**Authors:** Bin Bai, Zimeng Li, Hongmei Wang, Xiaolin Du, Ling Wu, Jiuyuan Du, Caixia Lan

**Affiliations:** ^1^Wheat Research Institute, Gansu Academy of Agricultural Sciences, Lanzhou, China; ^2^Hubei Hongshan Laboratory, College of Plant Science and Technology, Huazhong Agricultural University, Wuhan, China; ^3^Institute of Biotechnology, Gansu Academy of Agricultural Sciences, Lanzhou, China; ^4^College of Life Science and Technology, Gansu Agricultural University, Lanzhou, China; ^5^Crop Research Institute, Sichuan Academy of Agricultural Sciences, Chengdu, China

**Keywords:** genetic mapping, quantitative traits, adult plant resistance, stripe rust, wheat

## Abstract

Wheat stripe rust is an important foliar disease that affects the wheat yield globally. Breeding for resistant wheat varieties is one of the most economically and environmentally effective ways to control this disease. The common wheat (*Triticum aestivum* L.) cultivar “Pascal” exhibited susceptibility to stripe rust at the seedling stage but it showed high resistance to stripe rust at the adult plant stage over 20 years in Gansu, a hotspot of the disease in northwestern China. To understand the genetic mechanism of stripe rust resistance in this cultivar, a 55K SNP array was used to analyze the two parents and the 220 recombinant inbred lines (RILs) derived from the cross of “Huixianhong” × “Pascal.” We detected three new stripe rust adult plant resistance (APR) quantitative trait locus (QTL) contributed by Pascal, viz. *QYr.gaas-1AL*, *QYr.gaas-3DL*, and *QYr.gaas-5AS*, using the inclusive composite interval mapping method. They were flanked by SNP markers *AX-111218361*—*AX-110577861*, *AX-111460455*—*AX-108798599*, and *AX-111523523*—*AX-110028503*, respectively, and explained the phenotypic variation ranging from 11.0 to 23.1%. Bulked segregant exome capture sequencing (BSE-Seq) was used for fine mapping of *QYr.gaas-1AL* and selection of candidate genes, *TraesCS1A02G313700*, *TraesCS1A02G313800*, and *TraesCS1A02G314900* for *QYr.gaas-1AL*. KASP markers *BSE-1A-12* and *HXPA-3D* for *QYr.gaas-1AL* and *QYr.gaas-3DL* were developed for breeders to develop durable stripe rust-resistant wheat varieties.

## Introduction

Wheat stripe rust, also known as yellow rust, caused by the air-borne fungus *Puccinia striiformis* f. sp. *tritici* (*Pst*), is considered the primary biotic threat to wheat production globally (Todorovska et al., 2009). In 2017, 88% of the world’s wheat production was susceptible to stripe rust, causing yield losses estimated to be 1 billion USD (Brandt et al., 2021), and a countrywide epidemic affected about 5.5 million ha of wheat in China (Zeng et al., 2022). It shows regional characteristics and occurs frequently in the southwestern and northwestern of China, especially in the southeastern Gansu, a stripe rust hotspot in northwestern of China (Bai et al., 2012). The management of stripe rust includes the deployment of resistant wheat and the use of fungicide, and breeding resistant wheat cultivars is the most effective and economic strategy for controlling stripe rust (Wen et al., 2008).

Genetic resistance to stripe rust in wheat can be broadly divided into two phenotypically, mechanistically, and genetically distinct categories: (a) seedling resistance (all-stage resistance, ASR), which is detected at the seedling stage but is also expressed at all developmental stages. It usually shows a major effect and is race specific (Roberts and Caldwell, 1970; Lin and Chen, 2007); (b) adult plant resistance (APR), which is expressed at the adult plant stages and appears to be durable. Some APR genes are less characterized and are difficult to select phenotypically (Ren R. et al., 2012).

To date, most identified stripe rust resistance genes conferring “seedling resistance” encode classic nucleotide-binding site leucine-rich repeat (NBS-LRR) R proteins that recognize effectors and trigger a defense response to resist disease. While it is easy to overcome the recognition of a single classical R gene *via* a single genetic variation in an avirulence gene (Steuernagel et al., 2016; Schwessinger, 2017). The major epidemics causing severe economic losses have occurred in various regions of the world including United States, China, and Australia (Nsabiyera et al., 2018) due to virulence on seedling resistance genes including *Yr2*, *Yr6*, *Yr9*, *Yr17*, and *Yr27* (Wellings and McIntosh, 1990; Nsabiyera et al., 2018). Only a few seedling stripe rust resistance genes (i.e., *Yr5* and *Yr15*) are still effective to some *Pst* races in China, which drives the demand for persistent disease-resistant cultivars.

Adult plant resistance is generally known as more durable than ASR, since a single genetic variation appears insufficient to overcome this type of resistance in the asexual stage of *Pst* (Schwessinger, 2017). In general, APR delays infection and production of spore, leading to slow rusting phenotypes instead of complete immunity. This type of resistance gene usually encodes allele-specific protein variants that are molecularly unrelated to NBS-LRR proteins. In the case of these genes, *Yr18* and *Yr46* encode two types of transporters (Krattinger et al., 2009; Moore et al., 2015). In addition, *Yr36*, a chloroplast-localized kinase WKS1 regulating, reactive oxygen species production (Ellis et al., 2014; Gou et al., 2017). Since the durability of R genes is usually defined by the global genetic diversity of the pathogen, the combination of APR genes with ASR genes is the best way for a wheat breeder to develop the durable stripe rust resistant wheat variety at all plant stages. To date, more than 83 *Yr* genes or alleles (*Yr1*–*Yr83*) have been formally named in wheat (Li et al., 2020; Draz et al., 2021). Most of them are race-specific ASR genes, and only a few are APR genes. *Lr34/Yr18/Pm38/Sr57* (Dyck, 1991), *Lr46/Yr29/Pm39/Sr58* (Singh et al., 1998), and *Lr67/Yr46/Pm46/Sr55* (Herrera-Foessel et al., 2011) confer pleiotropic APR to stripe rust, leaf rust, powdery mildew, and stem rust. QTL analysis has been widely used to dissect complex traits through identifying the genomic location and effects of genes contributing to quantitative variation (Young, 1996). Over the past 20 years, more than 320 stripe rust resistance QTLs have been reported using different molecular markers, including diversity array technology (DArT), single sequence repeats (SSRs), and single-nucleotide polymorphisms (SNPs) (Lan et al., 2017); the genetic locations of these QTLs are continually being refined through fine mapping studies (Lan et al., 2017; Draz et al., 2021).

Various approaches can be used to identify genetic loci and genes in wheat. High-density SNP markers using gene-chip technology provide a superior approach for QTL mapping due to their high-throughput, efficiency, allele specificity, and high-resolution capacity (Yu et al., 2011; Draz et al., 2021). The Axiom wheat 55K SNP array with 53,063 SNP probes was selected from the wheat 660K SNP array, which is considered to be more appropriate for wheat genetic research (Ren et al., 2018; Huang et al., 2019). Based on the advantages of lower costs, higher accuracy, and its medium density, this SNP array has been widely used in QTL identification in wheat (Huang et al., 2019; Zhang et al., 2019). Also, whole-genome exome sequencing (WES) has been successfully applied for the identification of genetic loci and isolating genes in wheat (Henry et al., 2014; Mo et al., 2018). For instance, candidate natural variants were identified using a 110 Mb exome capture assay at the *Yr6* locus responsible for stripe rust resistance (Gardiner et al., 2016). A BSA-based exome capture sequencing pipeline for rapid gene cloning has been developed by Chengdu Tcuni Technology, which is an alternative method to effectively sequence coding regions with low cost in wheat (Dong et al., 2020). Therefore, SNP chip and WES approaches can be the effective strategies to find new genetic loci and candidate genes associated with important traits in wheat.

The Italian common winter wheat cv. Pascal (Lan Yin 1), introduced to Gansu Province of China in 1995, showed a high level of APR to stripe rust in the field but was susceptible to CYR32 and CYR33 at the seedling stage. However, the seedling gene locations and inheritance of APR to stripe rust in Pascal were not clear. Thus, the objectives of this study were to (1) map seedling stripe rust resistance gene in the Huixianhong/Pascal F_5_ RIL population; (2) identify the APR to stripe rust in the same population; (3) determine the interaction effect between the identified resistance loci on stripe rust in the adult plant stage; and (4) develop molecular markers of new and stable resistance QTL for the wheat breeder to breed durable stripe rust-resistant wheat variety.

## Materials and Methods

### Parent Materials

A population of 220 F_5_ RILs was developed from a cross between “Huixianhong” and “Pascal.” “Pascal” is a stripe rust-resistant parent, whereas the cultivar “Huixianhong” is highly susceptible to stripe rust in the field. Huixianhong was widely used as a susceptible spreader and negative control in the genetic analysis of wheat stripe rust resistance. A single seed descend method was used to develop the F_5_ RIL population.

### Seedling Test

The parents were evaluated for their reaction to stripe rust at the seedling stage using the *Pst* races CYR32 and CYR33 under greenhouse conditions at Huazhong Agricultural University, and the reaction to CYR34 was tested in the Gansu Academy of Agricultural Sciences. The races of the pathogen were inoculated by spraying urediniospores suspended in mineral oil using an atomizer when wheat seedlings reached the two-leaf stage. The inoculated plants were left in an open area for 20 min to facilitate the evaporation of the oil and then placed in the dew chamber of 7°C for 24 h and back to the greenhouse. The infection types (ITs) were recorded approximately 2 weeks post-inoculation, based on a 0–9 scale modified from McNeal et al. (1971), and ITs with the scale of “0, 1, 2, 3, 4,” “5, 6,” and “7, 8, 9” are categorized as resistant, intermediate, and susceptible groups, respectively.

### Adult Plant Stage of Field Test

The parents and RIL population were evaluated for APR to stripe rust in Qingshui Experimental Station of Gansu Academy of Agricultural Sciences during the 2017–2018 and the 2018–2019 growing seasons (GS2018 and GS2019), and in 2018–2019 growing season in Pidu Experimental Station of Sichuan Academy of Agricultural Sciences (SC2019).

Field plots consisted of 1.5-m rows planted with approximately fifty seeds of each line, and we used randomized complete block design with two replicates of the RIL population in each location. “Jinmai 47” was planted at every tenth plot of experimental materials and around the experiment as a susceptible check and spreader to ensure the maximum inoculum for disease development. Disease severity (DS) for the RILs and their parents in each environment was recorded one time or two times following the modified Cobb’s scale (Peterson et al., 1948).

In 2018, we took the first rating when the susceptible check Huixianhong showed approximately 80% DS around 12 June, and the second rating about a week later when the DS of the susceptible check reached 90–100% in Gansu. In the other two environments, we investigated the phenotype once when the DS of susceptible check reached 90–100%. Combined with the natural infection, a mixture of *Pst* races CYR32, CYR33, and CYR34 was used to inoculate the spreaders at the jointing growth stage (around 2 months after planting). The lme4 package in Rstudio^1^ was used to calculate the best linear unbiased prediction (BLUP).

### Correlation Analysis of the Phenotypic Data

SAS 9.2 software (SAS Institute, Cary, NC) was used to calculate the correlations of final disease severity (FDS) for stripe rust in each season. The analysis of variance (ANOVA) was used to assess the significant effect of a single stripe rust resistance QTL and the interaction effect for multiple stripe rust resistance QTLs with the FDS values in each environment.

### Genetic Linkage Map Construction and Quantitative Trait Locus Mapping

Deoxyribonucleic acid was extracted from about 20 plants of each line and parents using the cetyltrimethylammonium bromide (CTAB) method (Chatterjee et al., 2002). The 55K SNP array genotyping platform was used to genotype the parents and RIL population. A total of 55,000 SNP markers were obtained, of which 9,475 SNP markers were selected to make linkage groups by removing markers of distorted segregation (*p* < 0.001), monomorphic markers, and markers with more than 30% missing data. Genetic linkage groups were established using Joinmap 4.1 (Van, 2011) with a logarithm of odds (LODs) threshold of 10.0. MapChart (Voorrips, 2002) was used to draw genetic linkage maps. Software IciMapping 4.1 (Meng et al., 2015) was used to detect FDS-related QTL in each environment, BLUP and mean of final disease severity (MFDS) across each season to obtain the significant QTL position, LOD scores, phenotypic variance explained (PVE), and the additive effect of each locus. About one-thousand permutations were used to calculate LOD scores for each trait. The physical positions of identified QTL were compared with those previously reported on the same chromosome arm under different wheat lines, based on the wheat genome of (International Wheat Genome Sequencing Consortium [IWGSC], 2018).

### Bulked Segregant Exome Capture Sequencing

The bulked segregant exome capture sequencing (BSE-Seq) was used for identifying causal mutations or candidate genes in this study. First, the genomic DNA of 33 extremely resistance (low FDS) lines and 43 extremely susceptible (high FDS) lines from the Huixianhong/Pascal F_5_ population was extracted by CTAB method (Chatterjee et al., 2002) and then bulked in an equal amount of each line to generate the resistant-type bulked DNA pool and susceptible-type bulked DNA pool separately. The genomic DNA of Huixianhong and Pascal was also extracted using the same method as above. Therefore, the resistant-type bulked DNA pool, susceptible-type bulked DNA pool, resistant-type parent DNA, and susceptible-type parent DNA were used to Exome Capture Sequencing, which was developed by Chengdu Tcuni Technology.

The Euclidean Distance (ED) algorithm is a method using sequencing data to find significantly different markers between bulks and evaluates the region associated with the target trait (Hill et al., 2013).


$$E\,D=\sqrt{\begin{array}{c} {\left(A\,m\,u\,t-A\,w\,t\right)}^{2}+{\left(T\,m\,u\,t-T\,w\,t\right)}^{2}+{\left(C\,m\,u\,t-C\,w\,t\right)}^{2}\\ +{\left(G\,m\,u\,t-G\,w\,t\right)}^{2} \end{array}}$$

In the formula, A, T, C, and G mut are the frequencies of A, T, C, and G bases in the mutant bulk, respectively; A, T, C, and G wt are the frequencies of A, T, C, and G bases in the wild-type bulk, respectively. Theoretically, the ED values of other loci should tend to 0 except for the target trait-related sites between the two mixed pools.

## Results

### Phenotypic Analysis

**FIGURE 1:**
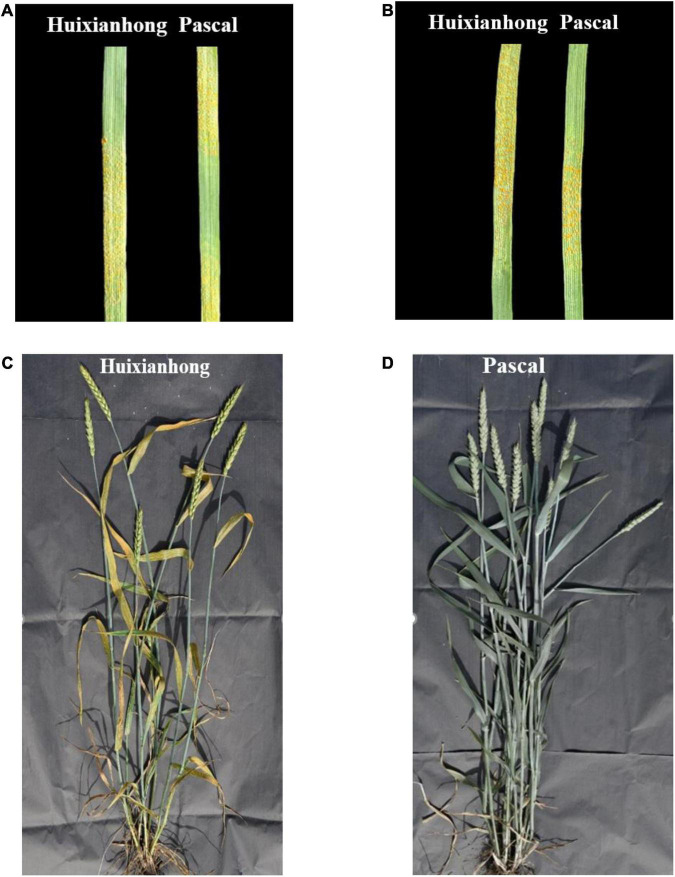
The two parents Huixianhong (left) and Pascal (right) showed susceptible against stripe rust race CYR32 **(A)** and CYR33 **(B)** at the seedling stage. The response of susceptible parent Huixianhong **(C)** and the resistant parent Pascal **(D)** in the adult plant stage under Gansu province field test.

**FIGURE 2:**
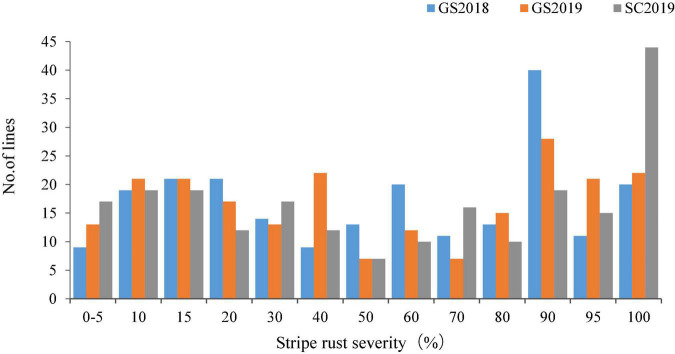
Frequency distributions of 220 Huixianhong/Pascal F_5_ recombinant inbred lines (RILs) for final disease severity of stripe rust in field traits at Gansu Province during 2017–2018 (GS2018), 2018–2019 (GS2019), and at Sichuan Province during 2018–2019 (SC2019).

Both Huixianhong and Pascal were susceptible after being inoculated by CYR32, CYR33, and CYR34 at the seedling stage (IT varied from 78 to 8) (Figures 1A,B). However, the MFDS for Huixianhong and Pascal were 100 and 3%, respectively, in the adult plant stage (Figures 1C,D) over three environments. The frequency distributions of stripe rust severity among F_5_ RILs showed a normal distribution (Figure 2), indicating a typical quantitative character for controlling stripe rust resistance in the Huixianhong/Pascal population. Pearson correlation coefficients (r) of stripe rust severity ranged from 0.66 to 0.85 over the environments (Table 1). In addition, it was estimated that there were around 3–4 stripe rust resistance genes with additive effects in the Huixianhong/Pascal RIL population based on the qualitative assessment of the Mendelian genetic segregation ratios (Table 2).

**TABLE 1:** Phenotypic Pearson’s correlations for stripe rust (GS2018, GS2019, SC2019) in the Huixianhong/Pascal F_5_ RIL population using the final disease severity for each season.

Environment	GS2018	GS2019	SC2019	YRM
GS2019	0.85**			
SC2019	0.66**	0.70**		
YRM	0.93**	0.91**	0.86**	
BLUP	0.91**	0.90**	0.87**	0.99**

GS2018, final stripe rust severity during Gansu 2017–2018; GS2019, final stripe rust severity during Gansu 2018–2019; SC2019, final stripe rust severity during Sichuan 2018–2019; YRM, the mean final disease severity of each season. **Significant at p < 0.0001.

**TABLE 2:** The number of resistance genes that confer adult plant resistance to stripe rust calculated by Mendelian segregation ratios in Huixianhong/Pascal recombinant inbred lines population.

Category	GS2018	GS2019	SC2019	YRM	BLUP
HTPR^a^	9	13	15	8	4
HTPS^b^	20	22	30	9	11
Other^c^	188	184	173	203	205
In total	217	219	218	220	220
No. of genes	3	3	3	4	4
*P*-value	0.08*	0.32*	0.01	0.95*	0.21*

*^a^HPTR means homozygous parental type resistant (RILs showing a similar phenotype as the resistant parent).*

*^b^HPTS means homozygous parental type susceptible (RILs showing a similar phenotype as the susceptible parent).*

*^c^Other means RILs showing different responses from the above two categories.*

*Means p > 0.05.

### Genetic Linkage Map Construction

**FIGURE 3:**
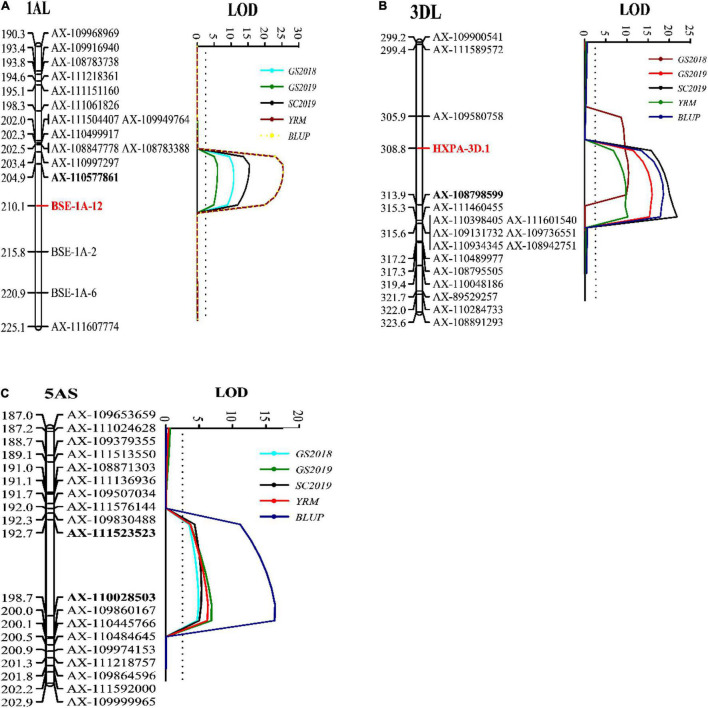
Likelihood plots of quantitative trait loci (QTL) for adult plant resistance to stripe rust on chromosomes 1AL **(A)** after adding three KASP markers *BSE-1A-2*, *BSE-1A-6*, and *BSE-1A-12*, 3DL **(B)** after adding KASP marker *HXPA-3D* and 5AS **(C)** identified by IciMapping 4.1 in the Huixianhong/Pascal RIL population. The significant LOD threshold was based on 1,000 permutations. Positions (in cM) of the molecular markers along chromosomes are shown on the vertical axes using cumulated genetic distances. KASP markers *BSE-1A-2*, *BSE-1A-6*, and *BSE-1A-12* near *QYr.gaas-1AL* were designed based on the SNP of BSE-Seq in the interval of original marker *AX-111218361—AX-110577861*. New marker *BSE-1A-12* and SNP marker *AX-110577861* were closely linked with *QYr.gaas-1AL* identified in the present study. we converted SNP marker *AX-109580758* as a KASP marker named *HXPA-3D* to detect 220 RIL families. KASP marker *HXPA-3D* was closely linked with *QYr.gaas-3DL* identified in the present study. GS2018, final stripe rust severity during Gansu 2017–2018; GS2019, final stripe rust severity, during Gansu 2018–2019; SC2019, final stripe rust severity during Sichuan 2018–2019; YRM, the mean final disease severity of each season; BLUP, best linear unbiased prediction.

A genetic linkage map was constructed with a total of 9,475 markers and developed 28 linkage groups on the 21 wheat chromosomes. A total of three QTLs were identified for stripe rust resistance from “Pascal” and they were mapped on wheat chromosomes 1AL (*QYr.gaas-1AL*), 3DL (*QYr.gaas-3DL*), and 5AS (*QYr.gaas-5AS*) (Table 3). *QYr.gaas-1AL* was flanked by SNP markers *AX-111218361* and *AX-110577861* and located at 505.3–507.9 Mb based on IWGSC RefSeq v1.0 genome sequence information. It was stably detected in all tested environments with PVE values ranging from 11.3 to 23.1% (Table 3). *QYr.gaas-3DL* was closely linked to markers *AX-108798599*, *AX-109580758*, and *AX-111460455*. It was located around 354.6 Mb based on IWGSC RefSeq v1.0 genome sequence information and was also consistently detected in all three stripe rust field experiments explaining 16.1–20.6% total stripe rust variation (Table 3). The third QTL, *QYr.gaas-5AS*, was flanked by SNP markers *AX-111523523* and *AX-110028503* and mapped at the interval of 59.1–62.2 Mb based on IWGSC 1.0 with PVE values ranging from 11.0 to 17.3% (Figure 3C and Table 3).

**TABLE 3:** Position and effect of quantitative trait loci (QTL) that were detected for adult plant resistance (APR) to stripe rust (YR) in the test years, using each final disease severity, the mean of final disease severity (MFDS), best linear unbiased prediction (BLUP) for the Huixianhong/Pascal RIL population.

QTL	Traits	Chr	Pos (cM)	Marker interval	Phy pos (Mb)	LOD^a^	PVE (%)^b^	Add^c^
*QYr.gaas-1AL*	GS2018	1A	202	*AX-111218361—–AX-110577861*	505.3–507.9	11.2	23.1	15.3
	GS2019	1A	202	*AX-111218361—–AX-110577861*	505.3–507.9	4.8	11.3	9.9
	SC2019	1A	202	*AX-111218361—–AX-110577861*	505.3–507.9	19.6	15.3	22.0
	YRM	1A	202	*AX-111218361—–AX-110577861*	505.3–507.9	27.2	16.2	19.9
	BLUP	1A	202	*AX-111218361—–AX-110577861*	505.3–507.9	27.2	15.9	17.3
*QYr.gaas-3DL*	SC2019	3D	350	*AX-111460455—–AX-108798599*	354.6–360.5	17.4	16.1	19.0
	YRM	3D	350	*AX-111460455—–AX-108798599*	354.6–360.5	18.4	20.6	15.9
	BLUP	3D	350	*AX-111460455—–AX-108798599*	354.6–360.5	18.1	20.3	13.8
	GS2018	3D	351	*AX-108798599—–AX-109580758*	339.7–354.6	13.1	16.4	15.3
	GS2019	3D	351	*AX-108798599—–AX-109580758*	339.7–354.6	15.5	17.5	17.4
*QYr.gaas-5AL*	SC2019A	5A	197	*AX-111523523—–AX-110028503*	59.1–62.2	5.4	11.0	12.8
	GS2018B	5A	198	*AX-111523523—–AX-110028503*	59.1–62.2	4.9	13.2	10.3
	GS2019B	5A	198	*AX-111523523—–AX-110028503*	59.1–62.2	6.9	15.7	12.6
	YRM	5A	198	*AX-111523523—–AX-110028503*	59.1–62.2	6.3	15.8	11.3
	BLUP	5A	198	*AX-111523523—–AX-110028503*	59.1–62.2	16.4	17.3	17.5

*^a^Logarithm of odds (LOD) score of QTL peak.*

*^b^Proportion of phenotypic variance explained by the QTL.*

*^c^Additive effect of phenotypic for each QTL.*

### Interactive Effects of Detected Resistance Loci

Based on the flanking molecular markers of detected three stripe rust resistance QTLs, we divided the F_5_ RILs into 8 groups. The presence of parental alleles for each QTL was determined by markers inferred factorial combinations of the three stripe rust resistance QTLs.

There was a significantly different disease response between RILs carrying either *QYr.gaas-1AL* or *QYr.gaas-3DL* and RILs without any resistance loci (*p* < 0.0001), which explained 24.2 and 19.3% of the stripe rust variation, respectively (Table 4). The lines carrying *QYr.gaas-5AS* also showed a significantly different disease response from those without any resistance locus, but this locus only explained 8.5% of the stripe rust variation. This indicates that this third locus imparts a minor effect on stripe rust resistance than the other two loci (Table 4). A significant interaction (*p* < 0.0001) between *QYr.gaas-1AL* and *QYr.gaas-3DL* was observed across three environments as well as MFDS, which explained 3.3% of stripe rust variation based on MFDS. There were no significant differences among the average FDS of RILs carrying combinations of QYr.gaas-1AL*Qyr.gaas-5AS, Qyr.gaas-3DL*Qyr.gaas-5AS, and QYr.gaas-1AL*QYr.gaas-3DL*QYr.gaas-5AS (*p > 0.05*); however, the average FDS of RILs carrying combinations of three resistance QTLs was significantly lower than the average FDS of RILs carrying other combinations of two resistance QTLs (*p < 0.0001*) (Table 4).

**TABLE 4:** Three-way factorial analysis of variance (ANOVA) of final disease severity (FDS) for stripe rust (YR) resistance loci, using years as blocks.

Source	No. of RILs	GS2018 FDS	GS2019 FDS	SC2019 FDS	MFDS^a^	DF	Type III SS	Mean square	*F*-value	Pr > F	Variation (%)
Year						2.0	1085.9	543.0	0.8	0.4	0.4
None	40.0	89.6A^b^	87.5A	80.9A	85.8A						
*QYr.gaas-1AL*	20.0	65.8BC	71.5AB	75.8A	70.8B	2.0	35459.6	17729.8	27.6	<0.0001	24.2
*QYr.gaas-3DL*	17.0	58.4BC	60.0BC	74.2A	61.5BC	3.0	41793.1	13931.0	21.7	<0.0001	19.3
*QYr.gaas-5AS*	20.0	67.4B	57.5BC	67.9A	66.4B	2.0	8755.1	4377.5	6.8	0.0	8.5
QYr.gaas-1AL *QYr.gaas-5AS	14.0	49.3CD	26.4D	45.6B	48.1C	2.0	5231.1	2615.5	4.1	0.0	0.5
QYr.gaas-1AL *QYr.gaas-3DL	31.0	38.8DE	31.4D	36.1BC	33.7D	4.0	19636.0	4909.0	7.6	<0.0001	3.3
QYr.gaas-3DL *QYr.gaas-5AS	7.0	33.2DE	17.7D	25.7C	27.4D	2.0	7184.2	3592.1	5.6	0.0	1.3
QYr.gaas-1AL *QYr.gaas-3DL *QYr.gaas-5AS	26.0	21.9E	21.5D	18.5C	20.4D	3.0	6082.8	2027.6	3.2	0.0	3.7

*^a^MFDS means the average of final disease severity over all tested environments, counted by the average YRM of RILs in each resistance genotype.*

*^b^Different letter in each columns means the significant difference between two values at p < 0.01.*

*Means the combination of two loci or three loci.

### Bulked Segregant Exome Capture Sequencing

**FIGURE 4:**
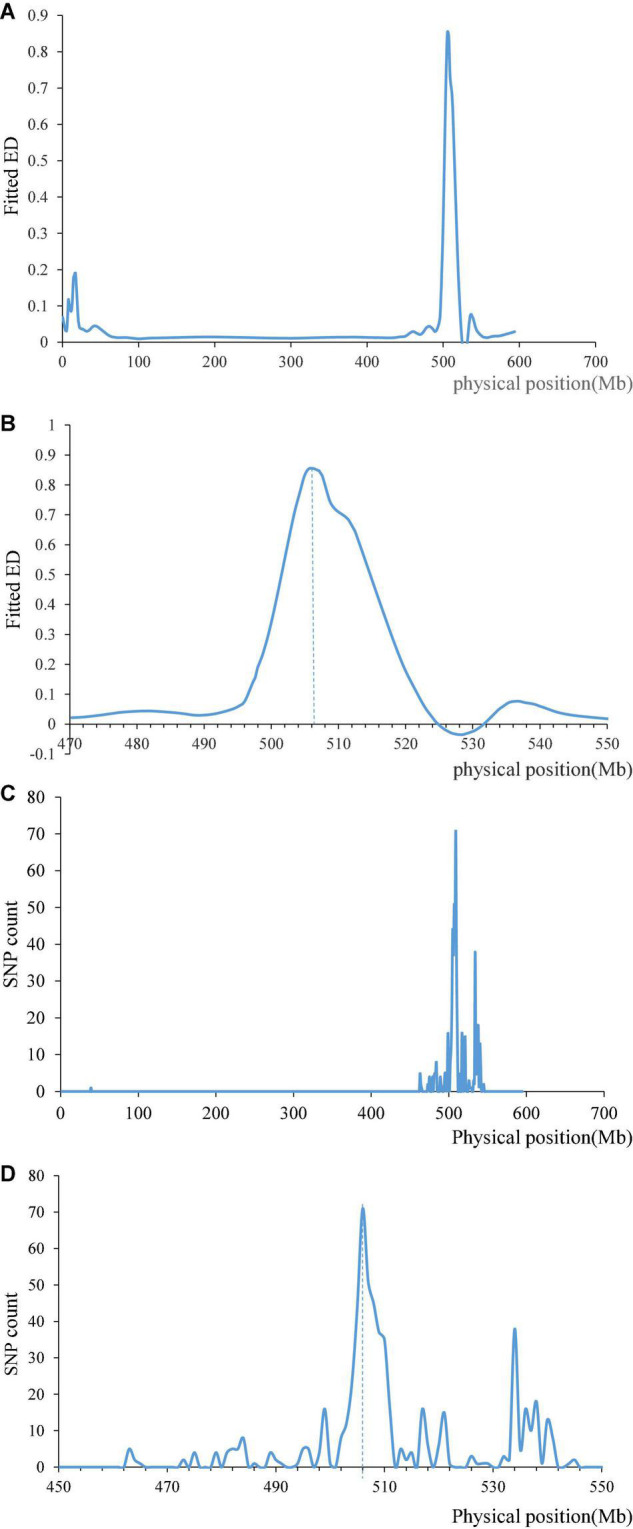
Results of the BSE-Seq analysis method. Fitted ED on chromosome 1A **(A)** and ED after zooming in the area near the maximum **(B)**. SNP count between R bulk same as Pascal and S bulk same as Huixianhong on chromosome 1A **(C)**, and SNP count after zooming in the area near the maximum **(D)**.

We filtered the data obtained by BSE-Seq with ref_Freq > 0.3, minDepth > 5 and ED = top 0.05 through the ED algorithm, and the results showed that there was a clear peak on chromosome 1AL, which is roughly around 506 Mb (Figures 4A,B). The SNPs between R bulk (same as resistance cultivar Pascal) and S bulk (same as susceptible cultivar Huixianhong) were filtered by AF < 0.2 or AF > 0.8, and the significant SNPs were found in a region of ∼506 Mb on chromosome 1A (Figures 4C,D). This result is also consistent with our mapping result, which indicates significant differences between two resistant and susceptible bulks and showed a high correlation between this region and stripe rust. Unfortunately, we failed to find high confidence SNPs on chromosome 3D through BSE-Seq, which may be related to the collection stage of materials or different process of related gene expression.

For fine mapping *QYr.gaas-1AL*, we developed three Kompetitive allele-specific PCR (KASP) markers near *QYr.gaas-1AL* based on the SNP of BSE-Seq. *QYr.gaas-1AL* was 0.5–3.5 cM away from KASP marker *BSE-1A-12* (Supplementary Table 1 and Figure 3A), which covered a physical interval of 505.3– 506.8 Mb on chromosome 1A. There were 18 high confidence genes in this interval, whereas 13 genes have SNPs on their exon regions. *TraesCS1A02G313700* has 14 SNPs on its exon regions which encodes a dentin sialophosphoprotein-like protein. *TraesCS1A02G313800* and *TraesCS1A02G314900* encode early light-induced protein and metacaspase, respectively, which have 13 and 10 SNPs on their exon regions. The three candidate genes might have strong relationship with *QYr.gaas-1AL.*

### The Distribution of *QYr.gaas-1AL* and *QYr.gaas-3DL*

Since *QYr.gaas-3DL* contributed stripe rust resistance in Pascal significantly, we converted SNP marker *AX-109580758* to a KASP marker and combined it with the phenotypic data for QTL mapping in 220 RIL families. The KASP marker *HXPA-3D* was 1.5–3.5 cm away from *QYr.gaas-3DL* (Figure 3B). This fact suggests that KASP marker *HXPA-3D* can be used as an effective marker to detect *QYr.gaas-3DL* for developing durable stripe rust resistant wheat varieties.

A total of two KASP markers *BSE-1A-12* and *HXPA-3D* for two stable loci were used to genotype a collection of 153 Chinese and global germplasm resources. In the global collection, *QYr.gaas-1AL* appears more frequently (69%), as compared to the Chinese wheat germplasm in which *QYr.gaas-1AL* appeared in 62%. Similarly, *QYr.gaas-3DL* is very common in foreign germplasm (75%), and it is significantly lower in Chinese wheat varieties and breeding lines (60%) (Supplementary Table 2). Our results suggest that Pascal could be a useful source of effective resistance against stripe rust in China.

## Discussion

“Pascal” was conferred by two recessive genes effective against two *Pst* races CYR 31 and CYR 32, respectively, at the seedling stage (Cao et al., 2006). In this study, we found both parents, Huixianhong and Pascal, susceptible to three *Pst* races of CYR32, CYR33, and CYR34 at the seedling stage (IT varied from 7 to 8). Most of the stripe rust resistance genes conferring seedling resistance can easily lose their effect since selection pressure favors the virulence in the pathogen population, which could explain that Pascal does not provide seedling resistance to stripe rust in this study. The constant evolution of pathogens and thus the breakdown of seedling resistance make our effort to continuously identify the sources of adult plant resistance increasingly important. We mapped three adult plant resistance QTLs for stripe rust in the Huixianhong/Pascal RIL population, and Pascal showed stable resistant over the last 50 years. There were five stripe rust resistance genes on chromosome 3D, viz. *Yr45* (Li et al., 2011), *Yr49* (Ellis et al., 2014), *Yr66* (Bariana et al., 2022), *Yr71* (Bariana et al., 2016), and *Yr73* (Dracatos et al., 2016). *Yr45*, *Yr71*, and *Yr73* were mapped on the 3DL, and the other two genes were mapped on 3DS. *Yr45* was discovered from a common spring wheat “PI181314,” flanked by RGAP markers *wgp118*, *wgp115*, and two SSR markers *Xwmc656* and *Xbarc6* which were mapped 11.7 and 12.6 cM proximal to *Yr45*. We tried to test the two parents using markers *wgp115*, *Xwmc656*, and *Xbarc6* (Li et al., 2011). However, none of them are polymorphic, and the physical interval of *Yr45* was from 476.9 to 514.9 Mb on chromosome 3D, whereas *QYr.gaas-3DL* was located around 354.6 Mb on the same chromosome. Besides, *Yr45* is an all-stage resistant gene, whereas *QYr.gaas-3DL* was an APR locus in wheat, and therefore, *Yr45* could be different from *QYr.gaas-3DL. Yr71* is an adult plant resistance gene mapped on 3DL in the Australian cultivar Sunco, and the physical position of its flanking marker *gwm114b* was at around 603.4 Mb (Bariana et al., 2016). KASP marker *Yr71_16434*, 1.8 cM distal to *Yr71*, was at 608 Mb on chromosome 3D. There was no polymorphic for *Xgwm114b* and *Yr71_16434A* in the “Huixianhong/Pascal” RILs population when we tried to detect the population, whereas *QYr.gaas-3DL* was located near the centromeric region of chromosome 3D. Another gene *Yr73*, a complementary gene on 3DL in the Australian cv. Avocet R, conferred seedling resistance to stripe rust. These results indicate that *QYr.gaas-3DL* is likely different from genes *Yr71* and *Yr73*.

The QTL *QYr/Lr.cim-3DC* for stripe rust near the centromeric region of chromosome 3D was identified in common wheat line UC1110 (Lan et al., 2017). It provided both of the leaf rust and stripe rust resistance in the population. *QYr.gaas-3DL* was also located near the centromeric region of chromosome 3D, but we did not detect the effect on leaf rust. *Qyrsicau-3DL*, a high-confidence APR locus was detected in a genome-wide association study (GWAS) in Sichuan wheat (Ye et al., 2019). The SNP marker *AX-109329567* closely linked with *Qyrsicau-3DL* was located at 595 Mb on chromosome 3D, which was about 240 Mb away from *QYr.gaas-3DL*. Huang et al. (2021) identified an APR QTL (*QYrsn.nwafu-3DL*) on 3DL explaining 5.8–12.2% of the phenotypic variation from Shannong 33 (SN33). The physical position of its flanking marker *AX-109582945* was around 407.4 Mb on chromosome 3D, which is around 52 Mb away from *QYr.gaas-3DL*. Furthermore, *QYrsn.nwafu-3DL* was present in 7.4% of a panel of 420 current Chinese wheat cultivars, which was much lower than the presence of *QYr.gaas-3DL* in a collection of 153 Chinese germplasm resources in our studies. Therefore, *QYrsn.nwafu-3DL* could be different from the APR resistance *QYr.gaas-3DL*.

The stripe rust resistance genes and QTL located on chromosome 5AS in the previous studies are extensive. A minor effect QTL (*QPst.jic-5A*) for field stripe rust resistance on chromosome 5AS was identified in a stripe rust susceptible winter wheat cv. Brigadier (Jagger et al., 2011). *QPst.jic-5A* was located between marker *Xwmc752* and *Xgwm786*, placing it near the centromere on the short arm of chromosome 5A. Since *QYr.gaas-5AS* was mapped at the interval of 59.1–62.2 Mb and far away from the centromere of chromosome 5A, *QPst.jic-5A* could be different from *QYr.gaas-5AS*. A major QTL, *QYr.cau-5AS*, exhibited 24.1–35.3% of PVE in winter wheat AQ24788-83 at the tillering growth stage (Quan et al., 2013). This QTL was flanked by markers *Xcfa2250* and *Xwmc705*, which were located around 245 Mb, suggesting this QTL was different from *QYr.gaas-5AS*.

*QYr.cim-5AS* was a minor APR QTL derived from Avocet and mapped near the SSR marker *Xcfa2104* and *Xbarc186* (Ren et al., 2017). The physical position of those SSR markers was around 22–46 Mb, placing near *QYr.gaas-5AS* in the present studies. However, it only explained 4.0–5.1% of YR variation, but *QYr.gaas-5AS* had PVE values ranging from 11.0 to 17.3%. It is possible that *QYr.gaas-5AS* is different from this QTL. However, this needs to be confirmed by allelism test in the future. *Qyrsicau-5AS*, a minor APR QTL with a PVE of 7.1–8.1%, were detected by the GWAS in Sichuan wheat (Ye et al., 2019). The SNP marker *AX-111623511* linked with *Qyrsicau-5AS* was located at 101 Mb on chromosome 5A, which was about 40 Mb from *QYr.gaas-5AS*, indicating *QYrsicau-5AS* could be different from the APR resistance *QYr.gaas-5AS*. Therefore, *QYr.gaas-5AS* is possibly a new QTL for APR to stripe rust.

Previous studies have detected several QTLs for stripe rust on chromosome 1AL in wheat. A minor APR QTL *QYr.sgi-1A* was located on chromosome 1A in the population of Kariega/Avocet S, contributing 6–12% to the phenotypic variance, and flanked by markers *s15m19D* and *s23m18E*, but was inconsistently detected across environments (Ramburan et al., 2004). Unfortunately, we failed to obtain the physical location of this QTL. Whereas *QYr.gaas-1AL* had PVE values ranging from 11.3 to 23.1%, it was stable across all environments in our studies, indicating that QTL *QYr.sgi-1A* was unlikely to be the same locus as *QYr.gaas-1AL*. Bariana et al. (2010) identified *QYr.sun-1A* controlling APR to stripe rust in Kukri/Janz-derived doubled haploid (DH) population, which explained 6–7% phenotypic variation for stripe rust. This QTL was flanked by marker *Xgwm164* and its physical position was around 280.6 Mb. But *QYr.gaas-1AL* was located in the interval of 505.3–507.9 Mb, which indicated that *QYr.gaas-1AL* was different from *QYr.sun-1A*.

Ren Y. et al. (2012) identified a QTL (*QYr.caas-1AL*) contributed APR to stripe rust in German spring wheat cultivar Naxos. It was located in marker interval *XwPt-2406–Xwmc59* at 575.4 Mb, which was roughly 70 Mb away from *QYr.caas-1AL*. Besides, *QYr.caas-1AL* only explained 8.2% of the phenotypic variance in one environment, which showed lower effect and stability than *QYr.gaas-1AL*. *QYrid.ui-1A*, a minor high-temperature adult plant (HTAP) resistance QTL from the hard red winter wheat germplasm IDO444, were significant only for IT in single environment (Chen et al., 2012). *QYr.gaas-1AL* had high effect of stripe rust resistance and were stable across all environments. Gebrewahid et al. (2020) identified an APR QTL on chromosome 1A in spring wheat Fuyu 3 designated *QYr.hebau-1AL*, which was located on the interval of *AX-109403007* to *AX-110502416* around 286.6–289.3 Mb. This QTL should be different from *QYr.gaas-1AL* based on a physical distance of about 220 Mb between them. Grover et al. (2022) identified an ASR locus *Yraci* in the European winter wheat cultivar “Acienda” on the distal end of wheat chromosome 1A. Besides, two SNPs *AX-95162217* and *AX-94540853* linked with *Yraci* were mapped in the bin at 54.04 cM on chromosome 1A, whereas *QYr.gaas-1AL* covered a physical interval of 505.3–506.8 Mb on chromosome 1A was far away from *Yraci*. Therefore, *Yraci* and *QYr.gaas-1AL* were not the same QTL for strip rust. Further research is needed to determine the novelty of *QYr.gaas-1AL* from Pascal.

Combining BSA and the exome sequence strategy could accelerate gene mapping, especially in wheat with a large and complex genome. BSE-Seq is helpful for the construction of a linkage map across the whole genome, and it could be easily used to identify the linked interval regardless of the multiple gene copies and obtain most of the variations existing in the coding regions of genes (Dong et al., 2020). Using bulked segregant analysis and the exome sequence strategy, Mo et al. (2018) identified a clear peak region on chromosome 4BS associated with increased plant height. Harrington et al. (2019) identified a locus controlling an environmentally dependent chlorosis phenotype in the Durum wheat cv. Kronos. Martinez et al. (2020) finely mapped a novel *TaMKK3* allele conferring the wheat ERA8 ABA-hypersensitive germination phenotype in a wheat backcross population. In our studies, BSE-Seq helped us to confirm the reality of QTL and select candidate genes in QTL *QYr.gaas-1AL* region. *TraesCS1A02G313700*, *TraesCS1A02G313800*, and *TraesCS1A02G314900* had more than 10 SNPs on their exon regions. Of these genes, *TraesCS1A02G314900* encodes metacaspase, which has been reported to modulate autophagy to confine cell death to the target cells during Arabidopsis vascular xylem differentiation (Escamez et al., 2016), which suggests that this gene may be a strong candidate related to disease resistance. Further research is needed to determine the relationship between these genes and stripe rust resistance.

## Data Availability Statement

The original contributions presented in the study are included in the article/Supplementary Material, further inquiries can be directed to the corresponding author/s.

## Author Contributions

BB initiated the project, designed the experiment, and contributed to phenotype data. CL designed the experiment and finalized the manuscript. ZL assisted in the data analysis and contributed to drafting the manuscript. HW and XD performed the sample preparation and DNA extraction. LW and JD contributed to the part of phenotype data in the field. All authors read and approved the final manuscript.

## Conflict of Interest

The authors declare that the research was conducted in the absence of any commercial or financial relationships that could be construed as a potential conflict of interest.

## Publisher’s Note

All claims expressed in this article are solely those of the authors and do not necessarily represent those of their affiliated organizations, or those of the publisher, the editors and the reviewers. Any product that may be evaluated in this article, or claim that may be made by its manufacturer, is not guaranteed or endorsed by the publisher.

## Footnotes

1
https://CRAN.R-project.org/package=lme4
