# Supplementary material for: Genetic Analysis of Adult Plant Resistance to Stripe Rust in Common Wheat Cultivar “Pascal”
Source: Front Plant Sci. 2022 Jul 6;13:918437. doi: 10.3389/fpls.2022.918437 (PMC9298664; doi:10.3389/fpls.2022.918437)
Supplement: Supplementary file 1 [file Data_Sheet_1.docx]

Supplementary Material

# Supplementary Table S1 Sequence and physical position of three KASP markers closely linked with *QYr.gaas-1AL* and variant feature of SNPs. SNPs were filtered by AF<0.2 or AF>0.8.

| Marker | POS (Kb) | REF | ALT | Feature | Marker-A | Marker-B | Marker-C |
| --- | --- | --- | --- | --- | --- | --- | --- |
| *BSE-1A-2* | 507,078,167 | G | A | missense_variant | ccctttgaGgacttcaGctC | ccctttgaGgacttcaGctT | tgctgaaTggtatggattcTgG |
| *BSE-1A-6* | 505,472,197 | C | T | missense_variant | agttgcttAgagtctccAacaacaC | agttgcttAgagtctccAacaacaT | cactttcagtctactggctcagAG |
| *BSE-1A-12* | 506,854,540 | A | T | missense_variant | cgcttttcaacTtaggatcctcaA | cgcttttcaacTtaggatcctcaT | tgctgcctcAtcaccgcA |

# Supplementary Table S2 Genotype of a collection of 153 Chinese and world important germplasm resources tested by KASP markers *BSE-1A-12* and *HXPA-3D* for two stable loci. No.1 to No.81 were Chinese materials, while No.82-No.153 were materials from abroad. “A” and “B” mean the genotype of these materials were the same as Huixianhong and Pascal, respectively, “H” means the genotype of these materials were heterozygous, “-” means the missing of genotype.

| **No** | **Cultivar** | ***BSE-1A-12*** | ***HXPA-3D*** |
| --- | --- | --- | --- |
| 1 | ZhongYou9507-1 | A | B |
| 2 | XinMai0401-2 | A | A |
| 3 | Ruihua1101-3 | A | - |
| 4 | ZhengYou27-2 | A | B |
| 5 | WenMai49-5 | A | A |
| 6 | XinMai23-2 | B | A |
| 7 | YuMai49-5 | B | B |
| 8 | HuaYu198-1 | A | B |
| 9 | ZhengNong16-1 | A | B |
| 10 | YuMai66-5 | B | H |
| 11 | ZhouMai22-2 | - | B |
| 12 | ZhengNong16 | B | B |
| 13 | AiKang58-5 | A | B |
| 14 | ZhengMai9023-3 | B | B |
| 15 | ZhuMai22-2 | A | A |
| 16 | ZhouMai20 | B | B |
| 17 | TaiXue7 | A | A |
| 18 | XuKe718 | B | B |
| 19 | YanGao1 | A | A |
| 20 | ShiMai15 | B | B |
| 21 | ZhouMai23 | B | B |
| 22 | ZhongMai175 | B | - |
| 23 | ZhongMai9-1 | B | A |
| 24 | ZhengMai9023 | B | A |
| 25 | YangMai12 | B | B |
| 26 | XuMai856 | B | A |
| 27 | GaoYou503 | H | A |
| 28 | YanNong999 | A | A |
| 29 | YuMai47 | A | B |
| 30 | YanMai98 | A | A |
| 31 | ZhongMai895 | A | B |
| 32 | ZhengNong16 | B | B |
| 33 | TaiShan21 | B | B |
| 34 | YanZhan1 | - | B |
| 35 | YuMai34 | B | B |
| 36 | TaiShan23 | B | A |
| 37 | EEn6 | B | A |
| 38 | HeNong972 | B | B |
| 39 | ShanNong28 | B | B |
| 40 | YanMai98 | A | A |
| 41 | YanGao21 | B | A |
| 42 | EEn1 | B | B |
| 43 | EMai12 | B | B |
| 44 | HeNong326 | A | B |
| 45 | Heng4338 | B | B |
| 46 | JinHe12-089 | B | B |
| 47 | JunMaiK8 | B | A |
| 48 | NanNong9918 | B | B |
| 49 | NeiMai9 | A | A |
| 50 | TaiShan23 | B | A |
| 51 | TaiShan24 | - | A |
| 52 | XiChang19 | A | B |
| 53 | XiKeMai6 | A | B |
| 54 | YangMai11 | B | B |
| 55 | YuMai34 | B | B |
| 56 | LuoYou7 | B | B |
| 57 | ShiMai13 | B | B |
| 58 | NongDa1193 | A | B |
| 59 | WanMai16 | B | B |
| 60 | ZhengMai119 | A | B |
| 61 | ZhengMai9023 | B | A |
| 62 | Sdau 22 | B | B |
| 63 | XinYou1 | B | B |
| 64 | EMai195（006） | B | H |
| 65 | E410187 | B | A |
| 66 | E610615 | A | B |
| 67 | ZhenMai9 | B | B |
| 68 | EMai21 | B | H |
| 69 | EMai039 | A | A |
| 70 | LuoMai36 | B | B |
| 71 | ShanNong102 | A | A |
| 72 | ZhongMai9-2 | A | A |
| 73 | ZhongYi6 | B | A |
| 74 | Luo6010 | B | B |
| 75 | EMai9721 | B | B |
| 76 | NingMaiZi1611 | B | B |
| 77 | LuoMai163 | B | B |
| 78 | 13213 | B | B |
| 79 | CuanMai65 | B | B |
| 80 | ChuanYu26 | B | H |
| 81 | ChuanMai 604 | B | A |
| 82 | ROLF07*2/DIAMONDBIRD//TRCH/HUIRIVIS #1/3/BORL14 | B | B |
| 83 | MEX94.27.1.20/3/SOKOLL//ATTILA/3*BCN/5/GK ARON/AG SECO 7846//2180/4/2*MILAN/KAUZ//PRINIA/3/BAV92 | B | A |
| 84 | MUNAL #1/7/CNO79//PF70354/MUS/3/PASTOR/4/BAV92/5/FRET2/KUKUNA//FRET2/6/MILAN/KAUZ//PRINIA/3/BAV92 | A | A |
| 85 | WHEAR//2*PRL/2*PASTOR/3/WAXBI/4/COPIO | A | B |
| 86 | NADI/3/PBW343*2/KUKUNA*2//FRTL/PIFED/4/NADI | B | H |
| 87 | ATTILA/3*BCN//BAV92/3/TILHI/4/SUP152/5/SUP152/6/KACHU #1/KIRITATI//KACHU | B | A |
| 88 | MUNAL*2//WAXWING*2/TUKURU/3/MUCUY | B | B |
| 89 | FITIS/3/KACHU #1/KIRITATI//KACHU | A | H |
| 90 | BORL14*2//MUNAL #1/FRANCOLIN #1 | B | B |
| 91 | MUU/5/WBLL1*2/4/YACO/PBW65/3/KAUZ*2/TRAP//KAUZ/6/WBLL1*2/SHAMA*2/7/PRL/2*PASTOR*2//FH6-1-7 | H | B |
| 92 | KIRITATI//PRL/2*PASTOR/5/OASIS/SKAUZ//4*BCN/3/PASTOR/4/KAUZ*2/YACO//KAUZ/6/KIRITATI//PRL/2*PASTOR/7/KSW/SAUAL//SAUAL/8/KIRITATI//PRL/2*PASTOR/5/OASIS/SKAUZ//4*BCN/3/PASTOR/4/KAUZ*2/YACO//KAUZ/6/KIRITATI//PRL/2*PASTOR | B | B |
| 93 | BABAX/LR42//BABAX*2/3/KUKUNA/4/CROSBILL #1/5/BECARD*2/6/KACHU/KINDE | B | B |
| 94 | BORL14/CHIPAK | B | B |
| 95 | CHIBIA//PRLII/CM65531/3/MISR 2*2/4/HUW234+LR34/PRINIA//PBW343*2/KUKUNA/3/ROLF07 | A | B |
| 96 | NELOKI//SOKOLL/EXCALIBUR | B | A |
| 97 | NELOKI//SOKOLL/EXCALIBUR | B | H |
| 98 | KASUKO | B | B |
| 99 | NELOKI//SOKOLL/EXCALIBUR | - | B |
| 100 | WBLL1*2/BRAMBLING//WBLL1*2/BRAMBLING/3/2*BORL14 | B | B |
| 101 | CIRO16/2*BORL14 | - | B |
| 102 | WBLL1*2/CHAPIO//HEILO/3/2*KSW/SAUAL//SAUAL | B | B |
| 103 | VILLA JUAREZ F2009/SOLALA//WBLL1*2/BRAMBLING/3/PBW343*2/KUKUNA*2//FRTL/PIFED | B | B |
| 104 | KOKILA/BOKOTA | A | A |
| 105 | VILLA JUAREZ F2009/3/T.DICOCCON PI94625/AE.SQUARROSA (372)//3*PASTOR/4/WBLL1*2/BRAMBLING/5/VALI/6/SUP152//WBLL1*2/BRAMBLING | - | B |
| 106 | ZINCOL/8/FRANCOLIN #1/7/REH/HARE//2*BCN/3/CROC_1/AE.SQUARROSA (213)//PGO/4/HUITES/5/T.SPELTA PI348599/6/REH/HARE//2*BCN/3/CROC_1/AE.SQUARROSA (213)//PGO/4/HUITES | B | B |
| 107 | WHEAR/KUKUNA/3/C80.1/3*BATAVIA//2*WBLL1/4/T.DICOCCON PI94625/AE.SQUARROSA (372)//SHA4/CHIL/5/WHEAR/KUKUNA/3/C80.1/3*BATAVIA//2*WBLL1/6/VILLA JUAREZ F2009/3/T.DICOCCON PI94625/AE.SQUARROSA (372)//3*PASTOR/4/WBLL1*2/BRAMBLING/7/TRAP#1/BOW/3/VEE/PJN//2* | - | B |
| 108 | DANPHE #1*2/3/T.DICOCCON PI94625/AE.SQUARROSA (372)//SHA4/CHIL/4/WBLL1*2/KURUKU//HEILO/5/WBLL1*2/KURUKU//HEILO | B | B |
| 109 | KVZ/PPR47.89C//TACUPETO F2001*2/BRAMBLING/3/2*TACUPETO F2001*2/BRAMBLING/4/KACHU/5/KACHU #1/3/C80.1/3*BATAVIA//2*WBLL1/4/KACHU | B | B |
| 110 | T.DICOCCON CI9309/AE.SQUARROSA (409)//2*PANDORA/5/WAXWING/3/BL 1496/MILAN//PI 610750/4/FRNCLN/6/KACHU/BECARD//WBLL1*2/BRAMBLING | B | B |
| 111 | SHAKTI/2*BORL14 | B | B |
| 112 | BORL14/FITIS | B | B |
| 113 | BORL14/FITIS | B | B |
| 114 | UP2338*2/KKTS*2//YANAC/3/WAXBI | B | A |
| 115 | BOKOTA//KFA/2*KACHU | B | B |
| 116 | VORB/FISCAL//WBLL1*2/KURUKU/3/QUAIU/4/KACHU/KINDE | B | B |
| 117 | PICUS/3/KAUZ*2/BOW//KAUZ/4/KKTS/5/T.SPELTA PI348530/6/2*FRANCOLIN #1/7/KACHU/KIRITATI | B | B |
| 118 | KACHU*2/SUP152 | B | B |
| 119 | KACHU*2/SUP152 | B | B |
| 120 | SAUAL/MUTUS//2*KACHU/KIRITATI | A | - |
| 121 | TACUPETO F2001*2/BRAMBLING//WBLL1*2/BRAMBLING/6/WBLL1*2/KURUKU*2/5/REH/HARE//2*BCN/3/CROC_1/AE.SQUARROSA (213)//PGO/4/HUITES/7/BAV92//IRENA/KAUZ/3/HUITES/4/2*ROLF07 | B | H |
| 122 | BOKOTA//BECARD/QUAIU #1/3/BOKOTA | A | A |
| 123 | FRET2*2/SHAMA*2/4/BOW/URES//2*WEAVER/3/CROC_1/AE.SQUARROSA (213)//PGO/5/KFA/2*KACHU/6/FRET2*2/SHAMA//KACHU | B | B |
| 124 | ATTILA*2/PBW65*2//TNMU*2/3/KFA/2*KACHU | A | B |
| 125 | BORL14*2//KFA/2*KACHU | A | B |
| 126 | BORL14*2//KFA/2*KACHU | A | - |
| 127 | WBLL1/FRET2//PASTOR*2/3/MURGA/6/KSW/5/2*ALTAR 84/AE.SQUARROSA (221)//3*BORL95/3/URES/JUN//KAUZ/4/WBLL1/7/KFA/2*KACHU | B | B |
| 128 | BAJ #1/3/KIRITATI//ATTILA*2/PASTOR*2/4/MUTUS*2/TECUE #1 | A | B |
| 129 | MUTUS/AKURI//SUP152/BAJ #1 | B | B |
| 130 | BAJ #1/AKURI//KACHU/KIRITATI | A | H |
| 131 | BORL14*2//KFA/2*KACHU | B | B |
| 132 | BORL14*2//BECARD/QUAIU #1 | B | B |
| 133 | SAUAL/YANAC//SAUAL/3/2*KUTZ | B | B |
| 134 | KUTZ*2//KFA/2*KACHU | A | B |
| 135 | BECARD #1/5/KIRITATI/4/2*SERI.1B*2/3/KAUZ*2/BOW//KAUZ*2/6/KFA/2*KACHU | B | B |
| 136 | KUTZ//KACHU/DANPHE | B | B |
| 137 | MUCUY//KACHU/KIRITATI | A | B |
| 138 | KACHU #1/3/T.DICOCCON PI94624/AE.SQUARROSA (409)//BCN/4/2*KACHU/5/SWSR22T.B./KACHU//2*KACHU | B | B |
| 139 | CHEWINK #1/FRNCLN/3/WBLL1*2/BRAMBLING*2//BAVIS | B | A |
| 140 | BAJ #1/TECUE #1//MUTUS*2/TECUE #1/3/MUCUY | B | A |
| 141 | KACHU #1//WBLL1*2/KUKUNA*2/6/BECARD #1/5/KIRITATI/4/2*SERI.1B*2/3/KAUZ*2/BOW//KAUZ | A | B |
| 142 | MUTUS*2/MUU//2*MUCUY | A | B |
| 143 | MUTUS*2/MUU//2*MUCUY | B | B |
| 144 | MUNAL/WESTONIA//2*BORL14 | B | B |
| 145 | FRET2/TUKURU//FRET2/3/MUNAL #1*2/4/KACHU/KIRITATI | B | B |
| 146 | BAJ #1*2/HUIRIVIS #1*2//TAITA | B | B |
| 147 | FRNCLN*2/BECARD//2*BORL14 | A | - |
| 148 | KASUKO | B | B |
| 149 | MUTUS//WBLL1*2/BRAMBLING/3/WBLL1*2/BRAMBLING/4/KFA/2*KACHU | B | - |
| 150 | KENYA SUNBIRD/KACHU//KACHU/KIRITATI | B | B |
| 151 | KACHU*2/3/ND643//2*PRL/2*PASTOR/4/KACHU/DANPHE | B | B |
| 152 | MANKU/6/WHEAR/KUKUNA/3/C80.1/3*BATAVIA//2*WBLL1/5/PRL/2*PASTOR/4/CHOIX/STAR/3/HE1/3*CNO79//2*SERI | B | B |
| 153 | WHEAR/KUKUNA/3/C80.1/3*BATAVIA//2*WBLL1/4/T.DICOCCON PI94625/AE.SQUARROSA (372)//3*PASTOR/5/WHEAR/KUKUNA/3/C80.1/3*BATAVIA//2*WBLL1/6/2*MANKU | B | B |
